# Quality of life following an open Latarjet-Bristow procedure in a general population with recurrent anterior shoulder instability

**DOI:** 10.1016/j.jseint.2022.12.003

**Published:** 2022-12-17

**Authors:** Collen S. Nkosi, Nyiko Z. Chauke

**Affiliations:** Chris Hani Baragwanath Academic Hospital, University of the Witwatersrand, Johannesburg, South Africa

**Keywords:** General population, Quality of life, Shoulder, Anterior instability, Latarjet-Bristow procedure, Recurrent dislocation

## Abstract

**Background:**

Despite the extensive literature on anterior glenohumeral instability, to date, there are no studies that report on the quality of life following a Latarjet-Bristow procedure with recurrent anterior shoulder instability. The purpose of this study was to evaluate the quality of life in patients who had a Latarjet-Bristow procedure.

**Methods:**

A single-center retrospective review with a prospective recall of patients who underwent a Latarjet-Bristow technique for recurrent anterior glenohumeral instability between January 2017 and March 2020. Outcomes measures included health-related quality of life using the Short Form-36 (SF-36) questionnaire and patient-related outcome measures using the Western Ontario Shoulder Instability Index and Rowe scores.

**Results:**

A total of 66 patients were identified to participate in the study; 40 (60.6%) responded and were included in the analysis. There were nine females and 31 males, with a median age of 32 years (27.5‒41 years). Three cases had bilateral anterior shoulder instability with a single joint being operated and three patients were epileptic. Physical and mental components summary of the SF-36 showed a better health-related quality of life in the general population. There was a significant strong correlation between SF-36 and Western Ontario Shoulder Instability Index. However, SF-36 and Rowe score showed a fair correlation.

**Conclusion:**

The Latarjet-Bristow procedure improves the quality of life in a general population similarly to an athletic population. The number of episodes of dislocation before surgery and the delayed surgical intervention did not increase the recurrent anterior shoulder instability rates postoperatively.

Glenohumeral joint instability is described as an abnormal movement of the shoulder joint secondary to disruption of either the static and/or dynamic stabilizer. Clinical glenohumeral instability is characterized by the pathomechanics of the injury, direction of instability, frequency of the instability episodes, and the severity of instability.^16^ Recurrence of the glenohumeral instability is the primary cause of disability among the teenage athletic population with a reported incidence of 24 cases/100 000 population per year.^8^^,^^19^^,^^25^ At least up to 90% risk of recurrent shoulder instability occurs within two years after the initial episode commonly in young males over 16 years old and females after age 70 years old, respectively.^8^^,^^25^^,^^27^

Management of the anterior glenohumeral instability has progressed over the past decades from nonsurgical to multiple surgical procedures techniques such as open procedures to a minimal invasive arthroscopic assisted surgery. Operative treatment can be grouped into soft tissue related and bony procedures or a combination of both procedures. The bony procedure and/or Bankart repair remains the gold standard procedure used in a patient with an osseous loss either at the glenoid, humeral head, and/or bipolar bone lesion.^4^^,^^10^^,^^12^^,^^20^^,^^24^^,^^26^ The Latarjet-Bristow procedure can either be performed arthroscopically or through open surgical techniques with equivalent clinical and functional outcomes.^17^^,^^24^ Clinical and functional outcomes are well published in the athletes and military population, however, few studies have reported in the general population improvement in the quality of life following a Latarjet or Bristow procedure.^10^^,^^17^^,^^20^

The purpose of the study was to evaluate the quality of life in patients who had a Latarjet-Bristow procedure. We hypothesize that the Latarjet-Bristow procedure improves the quality of life in a general population with recurrent shoulder instability similarly to published athletic population.

## Materials and methods

### Study design

A retrospective review with a prospective recall of patients who underwent a Latarjet-Bristow technique for recurrent anterior glenohumeral instability at the “C”: Chris Hani Baragwanath Academic Hospital Orthopedic Department between 01 January 2017 and 31 March 2020. The inclusion criteria were (i) patients treated with an open Latarjet-Bristow technique and (ii) patients ≥ 18 years old. The exclusion criteria were (i) patients with associated proximal humerus fracture, (ii) patients with a neurological deficit, (iii) patients with missing clinical records, and (iv) refusal to participate in the study.

Patients' admission preoperative clinical records, theater records, radiology department archives, and outpatient clinical records were reviewed. The data collected include patients' demographics, physical examination findings, radiographic findings, preoperative instability severity index score, patient-related outcome measures (Rowe score and Western Ontario Shoulder Instability index [WOSI]), and health-related quality of life (Short Form-36 [SF-36]) questionnaire.

### Statistical analysis

The data were collected, entered in Microsoft Excel (Microsoft, Redmond, WA, USA), and analyzed using the STATA software, version 15.2 (StataCorp, College Station, TX, USA). Descriptive statistics were used to summarize the results. Categorical variables were presented as frequencies and percentages, while continuous data were expressed as means and standard deviations suitable for normally distributed data and median and interquartile range (IQR) for nonnormal data. Correlation between two continuous variables was assessed using the Spearman correlation test on those variables, which were not normally distributed and the Pearson correlation test for those normally distributed. Normality was assessed using the Shapiro-Wilk test. The level of statistical significance was set at a *P* value of .05.

## Results

A total of 66 patients were identified and recalled for participation in the study; 40 patients responded and were included in the analysis. The total number of shoulders was 40 with three patients that sustained bilateral shoulder instability but unilaterally operated. The 26 patients who did not respond were excluded.

The majority of the patients were males consisting of 77.5% (*n* = 31), while females represented 22.5% (*n* = 9). The male-to-female ratio was 3.4:1. The median age of the patients was 32 years (IQR, 27.5 ‒ 41 years). The median age of males was 32 years and of females was 37 years. The majority 65% (*n* = 26) of the patients were younger than 35 years with a mean age of 28.5 years (range, 20‒35 years). According to our data, 77.5% (*n* = 31) of the patients were admitted with no comorbidities and 22.5% had the following comorbidities: 2.5% (*n* = 1) was type 2 diabetes mellitus, 2.5% (*n* = 1) was both hypertensive and epileptic, 5% (*n* = 2) were epileptic, 5% (*n* = 2) were hypertensive, and 7.5% (*n* = 3) had human immunodeficiency virus on treatment.

Recurrent anterior shoulder instability occurred more on the dominant side in 72.5% of the patients, and less common on the nondominant shoulder at 27.5%; however, three patients had bilateral shoulder instability. The three patients with bilateral shoulder instability were all unilateral operated on the more unstable shoulder, two had their dominant side operated, and one had the nondominant side operated. The right-hand side was involved in 60% (*n* = 24) of the patients and the left-hand side was less involved with 32.5% (*n* = 13), and bilateral shoulder involvement occurred in 7.5% (*n* = 3). Half of the patients were involved in some recreational sports activities mainly soccer 50% (*n* = 20). Two (5%) patients were professional rugby players, one patient played in the provincial league, and the second patient played in the tertiary league. The remaining 45% of the patients did not play sports.

In this study, 62.5% of the patients had more than five recurrent anterior shoulder dislocations before they had surgery and 37.5% patients had only one episode of anterior shoulder instability. More than half (55%) of the patients had their surgery within three years. Fifteen percent (15%) of the patients were operated between 4 to 6 years, 12.5% of the patients were operated between 7 to 9 years, and 17.5% had their surgery at or after the 10th year. The mean waiting period before surgery was 5.08 years (range: 0‒31 years). The overall mean follow-up for the fourth patients included in this study was 26.1 months (range: 10‒47 months).

### Preoperative instability severity index score findings

The preoperative instability severity index score (ISIS score) mean was 4.6 (range 4‒10) and the median was 4 (IQR: 4‒5). Preoperative Hill-Sachs and bony Bankart lesions dominated the scores causing higher ISIS scores.

### Postoperative health-related quality of life system findings

The SF-36 questionnaire was completed by all 40 patients on review. They revealed a mean physical component summary (PCS) of 72.78 compared to the mental component summary (MCS) mean of 68.04.

### Postoperative Western Ontario Shoulder Instability Index findings

Physical symptoms domain scored an excellent mean of 37.35% (standard deviation [SD]: +/- 14.11). Lifestyle and emotion domains had a good mean of 63.68% (SD: +/-18.17) and 69.85 (SD: +/-22.37), respectively. Sport/recreation/work domain had a fair mean of 72.48% (SD: +/-19.31). The WOSI total mean score was good at 53.67%.

### Postoperative Rowe score findings

The Rowe total score postoperative demonstrated good results with a mean of 78.25 (SD+/-20.89) and an excellent median of 90 (interquartile: 70‒90). There were more patients with excellent results (60%), followed by fair results at 20%, good results at 10%, and poor results at 10%.

### SF-36, Rowe score, and WOSI score correction findings

There was one significant correction between vitality SF-36 subscale and Rowe score (*P* = .0215). The other subscales of the SF-36 were mildly significant when correlated with the Rowe score. There were significant correlations between WOSI and SF-36 subscales; role limitations due to emotional problems (*P* = .0017), vitality (*P* = .0497), mental health (*P* = .0059), social functioning (*P* = .0264), and general health (*P* = .0159). There was a slight correlation significant between Rowe and WOSI scores (*P* = .3108).

### Number of shoulder instabilities with scoring instruments

Patients were grouped into three groups according to the number of instabilities they had prior to surgery (see Table I). The ISI score mean values (4.3 and 4.4) were similar in groups one and three, and slightly increased in group two with a mean of 5 points. The Rowe score had good results with all three groups scoring a mean above 75. The WOSI had similar moderate results among the groups scoring a mean above 50%. The SF-36 PCS and MCS showed excellent results scoring a mean above 70 in all the groups, except group two scored below 70 on MCS with good results.

**Table I:** Summary of number of shoulder instabilities with scoring instruments.

Items	Subscales	Group 1	Group 2	Group 3
Instability number		1-5	6-10	>11
Number of cases		15 (37.5%)	16 (40.0%)	9 (22.5%)
				
		Mean (SD)	Mean (SD)	Mean (SD)
ISI score		4.3 (0.46)	5 (1.55)	4.4 (0.53)
Rowe		75.0 (23.45)	81.6 (14,34)	77.8 (27.17)
WOSI		53.7 (11.60)	53.9 (13.69)	53.3 (18.00)
				
SF-36	Physical functioning	83.0 (11.92)	85.6 (10.93)	86.1 (11.39)
	Role limitations due to emotional problems	64.2 (49.72)	62.5 (50.00)	55.6 (52.70)
	Bodily pain	72.3 (31.65)	89.3 (12.39)	90.8 (18.00)
	General health	69.3 (18.11)	69.4 (22.12)	75.6 (18.44)
	PCS	72.2 (27.85)	76.7 (23.86)	77.0 (25.13)
	Role limitations due to physical health	68.3 (35.93)	42.2 (45.38)	80.6 (30.05)
	Vitality	63.3 (14.84)	65.3 (15.21)	66.7 (19.04)
	Mental health	70.6 (21.52)	71.3 (14.10)	69.3 (14.42)
	Social functioning	82.5 (23.71)	88.9 (18.16)	75.0 (35.35)
	MCS	71.18 (24.00)	66.93 (23.21)	72.9 (24.72)

*PCS*, physical component summary; *MCS*, mental component summary; *SD*, standard deviation; *SF-36*, Short Form-36; *WOSI*, Western Ontario Shoulder Instability Index; *ISI score*, instability severity index score.

## Discussion

The overall mean age at surgery was 34.2 years (range: 20–55). This study's general population age group was not different compared to studies with the athletic population. Hurley et al in a long-term outcomes study with athletes had a mean age of 27.4 years (range: 15‒58 years) at the surgery.^14^ In this study, athletes mean age was 22.5 years (range: 20‒25 years) at surgery. Baverel et al^2^ study had almost half (46.2 %) of the study population being recreational athletes with a mean age of 22.5 years (16‒29.3 years). In this study, the general population consisted more of (*n* = 20, 50%) recreational activities participants with a mean age of 31.6 years (range: 22‒49 years).

There were 31 (77.5%) men and 9 women (22.5%) in this study. Manual laborer contributed 16 (40%); 14 (35%) males and 2 (5%) females. The majority of the manual laborers were male (10/16–63%). There were two (5%) professional rugby players, both males. Compared to other previous studies, male gender was commonly operated on with recurrent anterior shoulder instability.^4^^,^^6^^,^^14^^,^^21^^,^^28^^,^^29^

In this study, three cases were epileptic with one case experiencing re-dislocation due to uncontrolled disease and revision surgery was delayed due to uncontrolled disease. In a systematic review Cowling et al reported on two studies with epileptic cases with recurrent shoulder instability one study had 2 re-dislocation cases of 30 cases in the study and Helfet reported 6 re-dislocation cases of 14 cases in the study.^10^^,^^12^ The Latarjet-Bristow procedure should be performed on well-controlled cases with epilepsy with recurrent anterior shoulder dislocation.^11^ All patients with human immunodeficiency virus and diabetes mellitus in this study were on treatment preoperatively and they did not experience wound healing issues. The majority of the patients in this study (75%) had no comorbidities, while Shah et al, in their study, had 88% with no comorbidities.^27^ Epilepsy altered health-related quality of life of one patient and the same patient had a poor Rowe score of 15. However, the overall health-related quality of life was not affected by comorbid diseases.

In our study, the dominant arm was commonly involved in 72.5% of the cases higher than the study reported by Boileau et al who had 64% of the dominant arm involved.^5^ Shah et al and Liu et al reported an equal occurrence of instability between nondominant and dominant extremities.^29^^,^^30^ Longo et al reported dominant-side involvement in 59.1% of their patients. In our study, 60% (24) of patients had right-sided instability while 7.5% (3) had bilateral involvement. In contrast, Hovelius et al reported that bilateral shoulder instability can be as high as 25% in patients of different ages.^13^

The incident of instability in this general population was more among none sports (45%) and soccer recreational athletes (50%), and less common on professional athletes (5%). Trauma-related shoulder dislocation was the primary cause of recurrent anterior shoulder instability with 95% of cases. Shah et al reviewed 22 rugby players with traumatic anterior glenohumeral instability.^22^ Seventy-five percent (75%) of anterior shoulder instability occurs during sports activities.^30^ In this study, only 5% of the population were sports players but with a similar cause of trauma-related injury. Shah et al reported trauma to be 80-97% cause of traumatic anterior shoulder instability.^27^ Stirma et al reviewed 12 to 10 professional soccer players with trauma related recurrent anterior shoulder instability.^31^

Majority of the patients (62.5%) in this study had more than five instabilities, and 30% had to wait for more than six years to get their surgery. Shoulder instability surgery has become more popular and is often done after the first shoulder dislocation in athletes.^27^ In our study, the mean wait before surgery was 5.1 years (range: 0–31). Du Plessis et al reported in their study that the delay of surgery did not influence their clinical outcomes.^11^ Stirma et al with 12 to 10 cases of professional football (soccer) players who had a 12.4-month waiting period prior to surgery and a mean of 4.3 dislocations prior to surgery had excellent clinical outcomes with no complications.^31^ The time to surgery and number of shoulder instability did not influence the health-related quality of life and patient related outcome measures of the patients included in this study.

In this study the ISIS median was 4 with a mean of 4.6. In their study Balg et al recommended an open Latarjet procedure for patients with an ISIS of 6 or greater to minimize recurrence as it was 70% when performed arthroscopically,^1^ while Boileau et al^7^ recommended arthroscopic surgery with an ISIS of less than 3 points and open surgery if 3 or more. The ISIS may still be relevant for institutions without preoperative computed tomography scan and patients with recurrent anterior shoulder instability indications. We support the study by Boileau et al^7^ and recommend the use of 3 points or more as a preoperative indication.

On the mental health domains, vitality, MCS, and role emotion had slight impairment with social functioning and mental health domains demonstrating greater results. The bodily pain score was high implying that pain was not a factor affecting health-related quality of life in this general population. The physical functioning score was very high which demonstrated excellent health-related quality of life functioning results for the general population with instability. PCS and MCS in our study showed means of 72.8 and 68.0 (Table II). In other orthopedic surgery conditions, Chung et al had a series of arthroscopic rotator cuff repair with 309 cases that were followed up for 26.3 months (range: 12–48) and had an improved PCS and MCS at final follow-up of 48.24 and 50.45.^9^ Their study represents a lower PCS and MCS at similar follow-up with our study but also with significant improvement at follow-up. In a retrospective study Leite et al had 35 cases who had undergone reverse shoulder arthroplasty and showed health-related quality of life with mean PCS of 50.01 and MCS of 54.05.^18^ Our data demonstrated a better health-related quality of life at a mean 26.1 months follow-up, with all the mean subscale scores higher than 60.

**Table II:** Summary of SF-36 findings.

	Subscales	Mean (SD)	Median (IQR)
SF-36	Physical component summary (PCS)		
	Physical functioning (PF)	84.75 (11.21)	90 (80-92.5)
	Bodily pain (BP)	75.05 (26.02)	77.5 (63.75-100)
	General health (GH)	70.75 (19.56)	75 (60-80)
	Role limitations due to physical health (RP)	60.6 (41.17)	75 (12.5-100)
	Mental component summary (MCS)		
	Mental health (MH)/Emotional well-being	70.6 (16.91)	72 (62-84)
	Social functioning (SF)	75 (27.15)	87.5 (50-100)
	Role limitations due to emotional problems (RE)	61.67 (48.65)	100 (0-100)
	Vitality (VT)/Energy/fatigue	64.88 (15.63)	65 (50-75)

*SD*, standard deviation; *IQR*, interquartile range; *SF-36*, Short Form-36.

Our postoperative mean WOSI score was 53.7% with one domain scoring excellent results: physical symptoms - 37.4%. Physical symptoms and lifestyle domains demonstrated satisfactory outcomes. Baverel et al retrospectively reviewed 106 athletes after an open Latarjet comparing 57 competitive athletes to 49 recreational athletes with a mean follow-up of 46 months. They had a postoperative mean WOSI score of 196.4 in competitive athletes and 357.7 in recreational athletes with an overall average of 277.1.^2^ Their study only had athletes but demonstrated excellent results compared to our study of the general population; however, Belangero et al in a prospective study with 37 athletes (41 shoulders) compared Latarjet and Bristow procedures with 5 years follow-up. Their two groups had mean WOSI scores of 54.0 and 52.6 demonstrating similar results to our study.^3^

The Rowe functional outcome score in our study was graded as poor (score 50 or less) preoperatively due to anterior shoulder instability with all our patients sustaining recurrent anterior shoulder instability. There were four (10%) patients postoperatively with poor Rowe results; three had recurrent anterior shoulder dislocation and one patient had severe osteoarthritis. There were eight (20%) patients with fair postoperative Rowe scores, which were affected by a positive apprehension test without instability in this group and they had scored 30 on stability criteria. Postoperatively, Rowe score total results were good with a mean of 78.25% (range: 10‒95%). Longo et al published a systematic review with 46 studies, of which 23 reported on functional outcomes using the Rowe score after an open Latarjet or Bristow, with a postoperative mean of 88.1.^20^ Baverel et al in a retrospective study of 106 athletes aged under 30 years reported improved Rowe scores between preoperative and postoperative analysis. Their postoperative Rowe scores were 82.2 and 69.5 for competitive athletes and recreational athletes, respectively.^2^ Our study showed a mean Rowe score of 78.3 with 60% of cases considered excellent and 20% of the cases considered good, similar to other published outcomes.

### SF-36, Rowe, and WOSI scores correlation

SF-36 subscales scores had a strong correlation with WOSI, five SF-36 subscales were statistically significant; however, the study by Kirkley et al showed a poor correlation between health measures and SF-12.^15^ SF-36 subscales scores had a fair correlation with the Rowe score, and two SF- 36 subscales were statistically significant. There was a mild correlation (0.310) between Rowe and WOSI scores similar to the study by Oh et all, in which the Rowe score was moderately correlated (0.471) with the WOSI for shoulder instability.^23^

### Number of shoulder instabilities with scoring instruments

We separated instability into three groups (see Table I). The number of instabilities per group prior to surgery did not affect the HRQoL, Rowe, and WOSI scores. The ISI score was mean 4 and above in the groups. The number of instabilities did not influence our outcomes similar to the study by du Plessis et al^11^ and Stirma et al.^31^

### Postoperative recurrent anterior shoulder dislocation

Three cases (7.5%) had recurrent anterior shoulder dislocation. This was similar to previously recurrent anterior shoulder instability reported postoperatively; Hurley et al^14^ had 8.5%, Bhatia et al^5^ reported 0-8%, and Cowling et al^10^ reported 5.36%. In this study, predisposing factors to postoperatively recurrent anterior shoulder instability were uncontrolled epilepsy, traumatic cause, and use of steroids.

## Conclusion

The Latarjet-Bristow procedure improves the quality of life in a general population similar to an athletic population. The number of episodes of dislocation or instability pattern and the delayed surgical intervention did not increase recurrent anterior shoulder instability rates.

## Disclaimers

Funding: No funding was disclosed by the authors.

Conflicts of interest: The authors, their immediate families, and any research foundation with which they are affiliated have not received any financial payments or other benefits from any commercial entity related to the subject of this article.

## References

[1] Balg F., Boileau P. (2007). The instability severity index score. A simple preoperative score to select patients for arthroscopic or open shoulder stabilisation. J bone Jt Surg Br.

[2] Baverel L., Colle P.E., Saffarini M., Anthony Odri G., Barth J. (2018). Open Latarjet procedures produce better outcomes in competitive athletes compared with recreational athletes: a clinical Comparative study of 106 athletes aged under 30 Years. Am J Sports Med.

[3] Belangero P.S., Lara P.H.S., Figueiredo E.A., Andreoli C.V., de Castro Pochini A., Ejnisman B. (2021). Bristow versus Latarjet in high-demand athletes with anterior shoulder instability: a prospective randomized comparison. JSES Int.

[4] Bessiere C., Trojani C., Carles M., Mehta S.S., Boileau P. (2014). The open Latarjet procedure is more reliable in terms of shoulder stability than arthroscopic Bankart repair. Clin Orthopaedic Relat Res.

[5] Bhatia S., Frank R.M., Ghodadra N.S., Hsu A.R., Romeo A.A., Bach B.R. (2014). The outcomes and surgical techniques of the Latarjet procedure. Arthrosc The J Arthroscopic Relat Surg.

[6] Boileau P., Gendre P., Baba M., Thelu C., Baring T., Gonzalez J. (2016). A guided surgical approach and novel fixation method for arthroscopic Latarjet. J Shoulder Elbow Surg.

[7] Boileau P., Lemmex D.B. (2019). Editorial commentary: which patients are likely to undergo redislocation after an arthroscopic Bankart repair? Preoperative instability severity index scoring over 3 points-the Game is over!. Arthroscopy.

[8] Brownson P., Donaldson O., Fox M., Rees J.L., Rangan A., Jaggi A. (2015). BESS/BOA patient care pathways: traumatic anterior shoulder instability. Shoulder and Elbow.

[9] Chung S.W., Park J.S., Kim S.H., Shin S.H., Oh J.H. (2012). Quality of life after arthroscopic rotator cuff repair: evaluation using SF-36 and an analysis of affecting clinical factors. Am J Sports Med.

[10] Cowling P.D., Akhtar M.A., Liow R.Y.L. (2016). What is a Bristow-Latarjet procedure? A review of the described operative techniques and outcomes. Bone Joint J.

[11] du Plessis J.-P., Lambrechts A., McGuire D., Roche S.J.L., Vrettos B.C. (2014). Early and medium-term complications of the modified Latarjet procedure. SA Orthopaedic J.

[12] Helfet A.J. (1958). Coracoid transplantation for recurring dislocation of the shoulder. J Bone Joint Surg (Britain).

[13] Hovelius L. (1999). The natural history of primary anterior dislocation of the shoulder in the young. J Orthopaedic Sci.

[14] Hurley E.T., Jamal M.S., Ali Z.S., Montgomentry C., Pauzenberger L., Mullett H. (2018). Long-term outcomes of the Latarjet procedure for anterior shoulder instability: a systematic review of studies at 10-year follow-up. J Shoulder Elbow Surg.

[15] Kirkley A., Griffin S., McLintock H., Ng L. (1998). The development and evaluation of a disease-specific quality of life measurement tool for shoulder instability. The Western Ontario Shoulder Instability Index (WOSI). Am J Sports Med.

[16] Kuhn J.E. (2010). A new classification system for shoulder instability. Br J Sports Med.

[17] Lafosse L., Boyle S., Gutierrez-Aramberri M., Shah A., Meller R. (2010). Arthroscopic Latarjet procedure. Orthopaedic Clin North Am.

[18] Leite L.M.B., Lins-Kusterer L., Belangero P.S., Patriota G., Ejnisman B. (2019). Quality of life in patients who have undergone reverse shoulder arthroplasty. Acta ortopedica brasileira.

[19] Leroux T., Wasserstein D., Veillette C., Khoshbin A., Henry P., Chanal J. (2014). Epidemiology of primary anterior shoulder dislocation requiring closed reduction in Ontario, Canada. Am J Sports Med.

[20] Longo U.G., Loppini M., Rizzello G., Ciuffreda M., Maffulli N., Denaro V. (2014). Latarjet, Bristow, and Eden–Hybinette procedures for anterior shoulder dislocation: systematic review and quantitative synthesis of the literature. Arthroscopy.

[21] Mizuno N., Denard O.J., Raiss P., Melis B., Walch G. (2014). Long-term results of the Latarjet procedure for anterior instability of the shoulder. J Shoulder Elbow Surg.

[22] Murray I.R., Goudie E.B., Petrigliano F.A., Robinson M. (2013). Functional anatomy and biomechanics of shoulder stability in the athlete. Clin Sports Med.

[23] Oh J.H., Jo K.H., Kim W.S., Gong H.S., Han S.G., Kim Y.H. (2009). Comparative evaluation of the measurement properties of various shoulder outcome instruments. Am J Sports Med.

[24] Randelli P., Fossati C., Stoppani C., Evola F.R., De Girolamo L. (2016). Open Latarjet versus arthroscopic Latarjet: clinical results and cost analysis. Knee Surg Sports Traumatol Arthrosc.

[25] Randelli P., Ragone V., Carminati S., Cabitza P. (2012). Risk factors for recurrence after Bankart repair a systematic review. Knee Surg Sports Traumatol Arthrosc.

[26] Scheibel M., Kraus N., Diederichs G., Haas N.P. (2008). Arthroscopic reconstruction of chronic anteroinferior glenoid defect using an autologous tricortical iliac crest bone grafting technique. Arch Orthopaedic Trauma Surg.

[27] Shah A., Judge A., Delmestri A., Edwards K., Arden N.K., Prieto-Alhambra D. (2017). Incidence of shoulder dislocations in the UK, 1995-2015: a population-based cohort study. Br Med J (Open).

[28] Shah A.A., Bulter B.R., Romanowski J., Goel D., Karadagli D., Warner J.J.P. (2012). Short-term complications of the Latarjet procedure. J Bone Joint Surg (America).

[29] Shah N., Nadiri M.N., Torrance E., Funk L. (2018). Arthroscopic repair of bony Bankart lesions in collision athletes. Shoulder and Elbow.

[30] Stephen H.L., Mark H.H. (1996). Anterior shoulder instability: current review. Clin Orthopaedics Relat Res.

[31] Stirma G.A., Lima E.B.S., Chaves D.H., Belangero P.S., Andreoli C.V., Ejnisman B. (2020). Latarjet procedure on anterior shoulder instability in professional soccer players. Acta ortopedica brasileira.

